# Toward a more systematic understanding of water insecurity coping strategies: insights from 11 global sites

**DOI:** 10.1136/bmjgh-2023-013754

**Published:** 2024-05-31

**Authors:** Shalean M Collins, Nancy Mock, M Pia Chaparro, Donald Rose, Benjamin Watkins, Amber Wutich, Sera L Young

**Affiliations:** 1 Department of International Health and Sustainable Development, Tulane University School of Public Health and Tropical Medicine, New Orleans, Louisiana, USA; 2 Department of Health Systems and Population Health, University of Washington School of Public Health, Seattle, Washington, USA; 3 Tulane Nutrition, School of Public Health and Tropical Medicine, Tulane University, New Orleans, Louisiana, USA; 4 School of Human Evolution and Social Change, Arizona State University, Tempe, Arizona, USA; 5 Anthropology, Northwestern University, Evanston, Illinois, USA; 1 Epidemiology, London School of Hygiene & Tropical Medicine, London, UK; 2 Arnold School of Public Health, University of South Carolina, Columbia, South Carolina, USA; 3 Anthropology, The Islamia University of Bahawalpur, Punjab, Pakistan; 4 University of Westminster, London, UK; 5 Anthropology, The University of North Carolina at Greensboro, Greensboro, North Carolina, USA; 6 Nutrition, University of North Carolina Chapel Hill, Chapel Hill, North Carolina, USA; 7 Geography, University of Miami, Coral Gables, Florida, USA

**Keywords:** Public Health, Cross-sectional survey

## Abstract

**Introduction:**

Water insecurity–the inability to access and benefit from affordable, reliable and safe water for basic needs–is a considerable global health threat. With the urgent need to target interventions to the most vulnerable, accurate and meaningful measurement is a priority. Households use diverse strategies to cope with water insecurity; however, these have not been systematically characterised nor measured. The Food Insecurity Coping Strategies Index has been insightful for targeting nutrition interventions to the most vulnerable. As a first step towards creating an analogous scale for water, this study characterises the largest empirical data set on water insecurity coping strategies and proposes guidance on measuring it using a novel toolkit.

**Methods:**

Open-ended responses on water insecurity coping (n=2301) were collected across 11 sites in 10 low- and middle-income countries in the Household Water InSecurity Experiences (HWISE) Scale validation study. Responses were characterised and compared with behaviours identified in the literature to construct an instrument to systematically assess coping.

**Results:**

We identified 19 distinct strategies that households used when experiencing water insecurity. These findings, paired with prior literature, were used to develop a Water Insecurity Coping Strategies Assessment Toolkit with guidance on its piloting to assess coping prevalence, frequency and severity.

**Conclusions:**

The widespread occurrence of water insecurity coping strategies underscores the importance of understanding their prevalence and severity. The Water Insecurity Coping Strategies Assessment Toolkit offers a comprehensive approach to evaluate these strategies and inform the design and monitoring of interventions targeting those most vulnerable to water insecurity.

**WHAT IS ALREADY KNOWN ON THIS TOPIC**

- Households and individuals employ a range of coping strategies in response to resource insecurity. The water literature has identified a need to better understand the prevalence, severity and frequency of water insecurity coping strategies to facilitate the development of an analogous tool to the Food Insecurity Coping Strategies Index.

**WHAT THIS STUDY ADDS**

- This first empirical study of coping, the results of which have been consolidated with the literature, provides an overview of the various behaviours that households use to ameliorate water insecurity. Based on these findings, we propose the Water Insecurity Coping Strategies Assessment Toolkit to systematise learning around coping.

**HOW THIS STUDY MIGHT AFFECT RESEARCH, PRACTICE AND OR POLICY**

- This work complements existing efforts to quantify experiences with water insecurity by outlining the behaviours that individuals and households engage in to achieve water security. Measuring coping will permit monitoring and evaluation of behaviours over time, enhancing our understanding of responses to water insecurity and allowing for more precise and targeted interventions.

## Background

Water is essential for daily needs, including consumption, hygiene, productive livelihoods^1^ and for social, cultural and religious functions.^2^ Globally, water crises present significant challenges to public health, with increasing and alarmingly high numbers of individuals experiencing severe water scarcity,^3^ excess^4^ and contamination.^5^ Recurrent water-related shocks, such as flooding^6^ and drought,^7^ are inextricably linked with and expected to worsen due to climate change.^8^ These co-occur with, and in some cases exacerbate, pollution of water resources^9–11^ and conflict.^12^ At the household level, water insecurity– the inability to access and benefit from affordable, adequate, reliable and safe water for well-being and a healthy life–has^13^ adverse economic, social, mental and physical health consequences and is a major contributor to morbidity and mortality.^14–20^

When households experience resource insecurity, they develop and employ behaviours to mitigate adverse outcomes.^21^ There are many ways to conceptualise and refer to these behaviours; here we use the term ‘coping strategies’. This is a phrase adapted from the psychology literature to mean voluntary behaviours used in response to exogenous stress.^22^ We distinguish coping from habituated behaviours (eg, routine household water management) by defining them as short-term responses driven by stress,^23 24^ such as severe resource deprivation or other shock.

Coping strategies have been widely studied since the 1990s in the context of income and safety net shocks,^25^ climate change^26^ and food insecurity.^27–29^ The suite of potential coping strategies is shaped by primary appraisal (ie, perceived harm caused by shocks) and secondary appraisal (ie, resources available to cope with shocks).^30^ As such, they vary widely by geography, culture, season, socioeconomic status and aetiology of resource insecurity. Despite this variation, food insecurity coping strategy measurement tools can rapidly generate information about the realities of a household’s situation, including documenting changes in resource availability and access over time^31^ coalescing around universal behaviours. These include intensification (eg, increasing availability, purchasing resources on credit, sending household members to beg for resources), modified consumption (eg, rationing supplies, relying on poorer quality resources), migration (eg, relocating to areas with more resources) and reprioritisation or abandonment (eg, withdrawing from sharing agreements, prioritisation of some household members over others).^32 33^

Although there has been robust theorisation about adaptation in response to water insecurity,^33^ empirical evidence of coping to support these frameworks is far more recent and limited in scope.^34–36^ The first systematic review of household water insecurity coping, published in 2016, found that the three most commonly documented strategies were drilling wells, storing water and collecting it from non-primary sources.^35^ A 2020 systematic review of coping strategies across 173 studies found that individuals and households improved their water availability by increasing storage capacity or building infrastructure, diversifying sources, purchasing or borrowing water, treating and consuming unsafe water and changing their routines to acquire water.^36^ In 2020, a meta-ethnographic synthesis of qualitative interviews identified nine coping strategies, some of which overlapped with those previously outlined. These including improving water storage capacity, constructing water sources, water management and reuse and collecting water from distant non-primary sources, among others.^34^ Although these reviews provide insight into how individuals and households cope with water insecurity, they may not sufficiently capture the panoply and severity of behavioural responses to water insecurity. This undermines our ability to create an analogous coping strategies index for water insecurity.^37^

### Objectives

Therefore, our first objective was to characterise household water insecurity coping strategies reported across a diverse range of low- and middle-income sites using the most comprehensive empirical data set available. The second was to build evidence on the relationship between coping and experiences of water insecurity. The third was to consolidate coping strategies using these data and the literature to create a Water Insecurity Coping Strategies Assessment Toolkit.

## Methods

### Study setting and data collection

Data were collected as part of the Household Water InSecurity Experiences (HWISE) Scale validation study (2017–2018),^38 39^ which implemented non-nationally representative cross-sectional surveys in 28 sites in 22 low- and middle-income countries in sub-Saharan Africa, Latin America and the Caribbean, the Middle East and Asia where water problems (eg, drought, flooding, chronic scarcity) had been documented. The primary objective of the HWISE study was to develop and validate a cross-context equivalent household water insecurity scale.^39^ As described elsewhere, sociodemographic and water-related data (eg, source, water insecurity, water borrowing practices, coping strategies) were collected from approximately 250 individuals who were knowledgeable about their household water situation in each site.^39^ This sample size was selected based on the minimum criteria required for scale validation.^39^

Sites were selected to maximise geographic, socioeconomic, ecological, seasonal and water infrastructure heterogeneity. Four sites used cluster randomised sampling (Morogoro, Tanzania; Beirut, Lebanon; Punjab, Pakistan; Labuan Bajo, Indonesia), three used stratified random sampling (Sistan and Baluchestan, Iran; Rajasthan, India; Chiquimula, Guatemala), two used simple random sampling (Torreón, Mexico and Cartagena, Colombia) and one (Gressier, Haiti) used case-base sampling.^39^ Data were collected using tablets programmed with Open Data Kit or paper forms translated into the languages identified as most commonly spoken in each of the sites. Interviews lasted approximately 45 min.

### Patient and public involvement

The initial development of the research questions was a result of qualitative work that has been documented elsewhere.^38^ Efforts detailed here used secondary data from that study for analyses, therefore it was not appropriate to involve the public in the design, conduct, reporting, or dissemination of this study.

### Coping strategy variable creation and coding

Coping strategies data were derived from one open-ended survey question ‘what do you do when you do not have enough water or money to buy water?’ asked mid-survey following a module on water insecurity and household water sources. Participants free-listed responses. This item was asked in 12 sites across Latin America and the Caribbean (Cartagena, Colombia; Chiquimula, Guatemala; Gressier, Haiti; Torreón, Mexico), sub-Saharan Africa (Morogoro, Tanzania), Asia (Punjab, Pakistan; Rajasthan, India; Labuan Bajo, Indonesia; Pune, India) and the Middle East (Beirut, Lebanon among Palestinian refugees and Lebanese Host Country Nationals; Sistan and Baluchestan, Iran) (table 1). Responses were translated into English by site study teams before analyses.

**Table 1:** Participant and site characteristics of HWISE coping sample (n=3063*; 2269†)

Site*	Reported at least one strategy, %	Mean (SD) HWISE Score	Age, mean (SD)	Female respondents, %	Site type	Season^39^	Primary water sources (%)
Torreón, Mexico (n=249; 137)	46.9	8.6 (8.4)	46.3 (16.6)	72.9	Peri-urban	Middle/end of dry season	Tanker truck (70.2); piped water (27.0)
Chiquimula, Guatemala (n=314; 294)	92.9	5.2 (5.3)	38.8 (14.9)	86.6	Peri-urban	Dry season	Piped water (64.9); protected spring (15.3)
Cartagena, Colombia (n=266; 246)	92.5	20.9 (7.5)	40.8 (15.1)	69.2	Urban	Dry season	Piped water (46.2); standpipe (34.6)
Gressier, Haiti (n=292; 205)	67.8	9.8 (9.1)	36.1 (13.9)	98.6	Rural/peri-urban	Dry season	Standpipe (26.8); rainwater (14.1)
Beirut, Lebanon (HCN) (n=375;138)	36.8	5.5 (6.5)	43.9 (15.4)	62.1	Urban	Rainy season	Bottled water (48.3); small water vendor (47.5)
Beirut, Lebanon (refugee) (n=199; 118)	59.3	10.2 (6.9)	40.9 (13.6)	66.8	Urban	Rainy season	Small water vendor (67.8); bottled water (23.6)
Sistan and Baluchestan, Iran (n=306; 296)	96.7	6.0 (6.5)	33.3 (10.9)	99.0	Urban	Rainy season	Rainwater (48.0); cistern (25.5)
Punjab, Pakistan (n=235; 126)	53.2	20.4 (5.9)	35.8 (10.1)	57.4	Urban	Dry season	Stand pipe (26.6); tubewell (23.2)
Rajasthan, India (n=248; 201)	81.0	13.9 (7.4)	41.9 (13.1)	27.0	Urban	Dry season	Small water vendor (55.2); tubewell (26.2)
Labuan Bajo, Indonesia (n=281; 266)	88.0	13.8 (7.7)	38.2 (11.3)	44.8	Urban	Dry season	Bottled water (36.9); Unprotected dug well (12.9)
Morogoro, Tanzania (n=300; 242)	80.3	4.2 (4.8)	40.1 (14.9)	78.3	Rural	Rainy season	Standpipe (70.7); other person (23.7)

*Number of HWISE respondents.

†Number of respondents who reported at least one coping strategy.

HWISE, Household Water InSecurity Experiences.

To the first objective of characterising coping strategies across a range of contexts, responses were analysed by importing all open-ended responses into Stata (Stata V.14, StataCorp, College Station, Texas). Most responses were one to two lines of text; any responses that indicated ‘not applicable’ or ‘I do not experience this’ were coded as ‘none’. Multipart responses, such as ‘we pray for rain or bring it from the nearby village’, were broken down into constituent observations, such that the unit of analysis was the coping strategy, not the number of respondents.

Responses were categorically coded based on an adapted qualitative framework developed by the lead author in a prior systematic literature review.^36^ The framework contained codes for spiritual and psychosocial coping, changing the quantity of water collected, reducing or modifying consumption of water or foods, collecting water from other sources, consuming water perceived to be unsafe, moving households or village, stealing water, earning money to buy water, fighting with other individuals to access water, storing water for later use, paying for water service, borrowing water, purchasing water outright or on credit, collecting and storing rainwater, improving or building infrastructure and going without water. During the coding process, additional themes, such as contacting service providers to restore service, sending children to collect water, and waiting for water were added. Responses were hand-coded using the ‘edit’ function in Stata, where a new variable was created, and similar coping strategies were grouped together. For example, ‘I borrow from my neighbour’ and ‘I borrow from my sister’ were operationalised as ‘borrow water’. All strategies with at least one response were included to capture the full range of behaviours.

### HWISE scores and sociodemographics

HWISE scores were calculated following standard procedures outlined elsewhere based on responses to 12 questions about their experiences with household water insecurity within the past 4 weeks.^38 39^ Response options were ‘never’ (0 times, scored as 0), ‘rarely’ (1–2 times, scored 1), ‘sometimes’ (3–10 times, scored 2)’, ‘often’ (11–20 times, scored 3) and ‘always’ (>20 times, scored 4). Responses for 'often' and 'always' were collapsed, and responses were summed (range 0-36), with higher scores indicating more experiences of water insecurity.^38 39^

Participant gender (female or male) was self-reported. Household location (ie, urban/peri-urban, rural, refugee camp) was determined by locally recruited enumerators. Seasonality was determined by local study teams.

### Statistical analysis

To the second objective, evaluating the relationship between coping and water insecurity, bivariate logistic regression models were built for each of the identified coping strategies with HWISE scores as the independent variable. Separate multivariable logistic regression models were built to determine the association between HWISE scores and each of the coping strategies. Models treated gender, rurality, season of data collection and primary water source as categorical predictors to approximate fixed effects to account for differences in sampling. Items with fewer than 10 responses were excluded from final analyses.

## Results

Across the 12 sites, 11 had sufficient data for the study; Pune, India was dropped for low response rate (n=2). Of the 3063 respondents across the 11 sites, 2269 reported at least one coping strategy, 31 respondents reported two strategies and one respondent reported three strategies, for a total of 2301 affirmative responses.

The majority (70.4%) of respondents were women, with a mean age of 39.6 years (SD 14.3); over half (61.5%) of households lived in urban or peri-urban areas (table 1). Approximately one-third of households (35.7%) were female-headed and most respondents were married or cohabiting (76.8%). Primary household drinking water sources varied across the sample (table 1); 24.6% relied on small water vendors or tanker trucks, 18.8% used piped water (public water supply networks), 17.1% used stand pipes and 12.2% used bottled water. More than half (67.6%) of households used an improved (ie, protected from external contamination)^40^ water source.

Site characteristics for the HWISE scale validation study^41^ have been detailed elsewhere.^39^ In the subsample included for this analysis, the mean (SD) HWISE score was 9.7 (±8.7) out of 36. Across sites, mean (SD) HWISE scores were highest in Cartagena, Colombia 20.9 (±7.5) and Punjab, Pakistan 20.4 (±5.9) and lowest in Morogoro, Tanzania 4.2 (±4.8) (table 1).

### Characterising coping strategies

Coping was reported by 74.3% of the total sample. Frequency of coping responses was highest in Sistan and Baluchestan, Iran (96.7%); Chiquimula, Guatemala (92.9%); Cartagena, Colombia (92.5%); Labuan Bajo, Indonesia (88.0%), Rajasthan, India (81.0%) and Morogoro, Tanzania (80.3%) (table 1).

Respondents reported 19 distinct coping strategies (table 2). Borrowing water was the most commonly reported strategy, practised by approximately half (50.8%) of respondents (table 2). Punjab, Pakistan and Chiquimula, Guatemala were exceptions, where borrowing was reported by 16.7% and 4.8% of respondents, respectively. Collecting water from a non-primary source was more commonly reported in both of these sites (table 2).

**Table 2:** Frequency of water insecurity coping across households in the Household Water Insecurity Experiences (HWISE) scale validation study (n=2301)

Coping strategies, %		Sub-Saharan Africa	Middle East		Asia					Latin America and the Caribbean		
	All sites (n=2301)	Morogoro, Tanzania (n=242)	Beirut, Lebanon (HCN) (n=138)	Beirut, Lebanon (Refugee) (n=118)	Sistan and Baluchestan, Iran (n=296)	Rajasthan, India (n=201)	Punjab, Pakistan (n=126)	Labuan Bajo, Indonesia (n=266)	Torreón, Mexico (n=137)	Cartagena, Colombia (n=246)	Chiquimula, Guatemala (n=294)	Gressier, Haiti (n=205)
Borrow water	50.8	72.3	43.5	50.0	93.9	64.2	16.7	52.6	36.5	71.9	4.76	30.2
Use non-primary source	24.2	15.7	2.17	0.85	0.00	19.9	42.1	41.4	3.65	11.4	81.3	13.2
Wait for water to return	3.78	0.41	0.00	0.00	0.00	0.00	5.56	0.00	22.6	6.10	0.00	15.1
Buy water on credit	3.21	1.24	31.2	6.78	0.00	0.00	0.79	0.00	2.19	1.22	0.00	5.37
Borrow money to buy water	5.87	1.65	18.8	40.7	6.42	8.96	0.79	0.38	0.73	4.88	0.00	2.44
Economise	3.00	1.65	1.45	0.85	0.00	3.48	0.00	0.75	6.57	4.47	8.50	2.93
Buy water	2.43	4.96	2.90	1.69	0.00	0.00	0.00	3.76	12.41	0.00	0.00	4.39
Use unsafe water	1.82	0.00	0.00	0.00	0.00	4.48	18.2	0.38	0.00	0.00	0.00	3.90
Do nothing	1.39	0.41	0.00	0.00	0.00	0.00	0.79	0.00	14.6	0.00	0.68	3.90
Psychosocial coping	1.35	0.00	0.00	0.00	0.00	0.00	0.79	0.00	0.00	0.81	0.34	13.2
Reduce or change consumption	0.74	0.41	0.00	0.00	0.00	0.00	0.00	0.38	0.73	0.81	3.40	0.98
Spiritual coping	0.48	0.00	0.00	0.00	0.00	0.00	5.56	0.00	0.00	0.00	0.00	1.95
Relocate or consider relocating	0.39	0.00	0.00	0.00	0.00	0.00	5.56	0.00	0.00	0.41	0.00	0.00
Work for water or money to buy water	0.30	0.41	0.00	0.00	0.00	0.50	3.17	0.00	0.00	0.00	0.00	0.49
Treat water	0.26	0.00	0.00	0.00	0.00	0.00	0.00	0.75	0.00	0.00	0.00	1.95
Go without water	0.26	0.83	0.00	0.00	0.00	0.00	0.00	0.00	0.00	0.00	0.68	0.49
Contact service provider	0.17	0.00	0.00	0.00	0.00	0.50	0.00	0.00	0.00	0.81	0.00	0.49
Repair source	0.04	0.00	0.00	0.00	0.00	0.00	0.00	0.00	0.00	0.00	0.34	0.00
Send children to collect water	0.04	0.00	0.00	0.00	0.00	0.00	0.79	0.00	0.00	0.00	0.00	0.00

Almost one-fourth of respondents (24.2%) coped with water insecurity by using non-primary sources (eg unprotected springs, streams, rivers) with poorer water quality than their documented primary sources (eg piped public water (35.3%); standpipes or tubewells (20.9%), small water vendors (9.4%); and protected springs (6.8%)). Forty-nine respondents traveled further from their homes to access alternative water sources due to unavailability of the primary source, and at least four respondents collected water early in the morning or in the evening.

Borrowing water and using an alternative source comprised the majority (75.0%) of coping responses. The other 17 strategies were far less common and included borrowing money to buy water (5.9%), waiting for water to return or for water supply to be restored (3.8%); economising or rationing water sources (3.0%); buying water (2.4%); using unsafe water (1.7%) and engaging in psychosocial coping such as complaining, resignation, suffering, anger, stress and despair and worrying (table 2). Three strategies were reported by fewer than 1% of respondents, including contacting a service provider, repairing a water source and sending children to collect water. Other notable but less frequently used strategies were reducing or changing consumption (eg, changing foods cooked, washing clothes in an open source instead of in household, rationing supplies) (0.74%), the use of spiritual coping (eg, prayer, relying on a deity to restore water) (0.48%), relocating or considering relocating households to be closer to a water source (0.39%), working for water or money to buy water (0.30%), treating water (0.26%) and going without (0.26%) (table 2).

#### Associations between coping strategies and HWISE scores

The second objective was building evidence on the relationship between coping strategies and water insecurity experiences. In multivariable models, households with higher HWISE scores had greater odds of borrowing water (OR 1.04, p<0.001), using water perceived to be unsafe (OR 1.13, p<0.001) and psychosocial coping (OR 1.06, p=0.01) and lower odds of using non-primary water sources (OR 0.96, p<0.001), economising (OR 0.96, p=0.04) and doing nothing (OR 0.91, p=0.001) (table 3).

**Table 3:** Bivariate and multivariable models of the relationship between between use of each coping strategy and water insecurity across 11 Household Water InSecurity Experiences (HWISE) scale validation study sites (n=2301)

Coping strategy^b^	HWISE score (0–36)	HWISE score (0–36) ^a^
	OR (95% CI)	OR (95% CI)
Borrow water (n=1170)	1.02 (1.02 to 1.04)***	1.04 (1.02 to 1.05)***
Use non-primary source (n=555)	0.97 (0.96 to 0.98)***	0.96 (0.94 to 0.98)***
Wait for water to return (n=87)	1.03 (1.00 to 1.05)**	0.98 (0.96 to 1.01)
Buy water on credit (n=74)	0.97 (0.95 to 1.00)	0.99 (0.96 to 1.03)
Borrow money to buy water (n=135)	0.97 (0.95-0.99)*	0.99 (0.97-1.02)
Economise (n=69)	0.98 (0.95 to 1.00)	0.96 (0.93 to 0.99)^*^
Buy water (n=56)	0.98 (0.94 to 1.01)	0.98 (0.95 to 1.02)
Use unsafe water (n=42)	1.08 (1.03 to 1.12)***	1.13 (1.07 to 1.19)***
Do nothing (n=32)	0.96 (0.91 to 1.00)	0.91 (0.87 to 0.96)**
Psychosocial coping (n=31)	1.05 (1.01 to 1.09)^*^	1.06 (1.01 to 1.12)^*^
Reduce or change consumption (n=17)	0.96 (0.89 to 1.02)	0.97 (0.90 to 1.04)
Spiritual coping (n=11)	0.98 (0.89 to 1.09)	0.92 (0.80 to 1.05)

Bolding indicates significance at either the p<0.05*, p<0.01**, or p<0.001*** levels.

^a^Controlling for respondent gender, season of data collection, rurality and primary water source.

^b^Each coping strategy was evaluated independently for its association with water insecurity, strategies were dichotomised to be yes/no responses. Treat water (n=6), go without water (n=6), contact service provider (n=4), relocating/consider relocating (n=9), repair source (n=1), send children to collect water (n=1) and work for water or money to buy water (n=7) were excluded due to insufficient sample size for analyses.

#### Consolidation of coping strategies

To the third objective of consolidating coping strategies into a Water Insecurity Coping Strategies Assessment Toolkit, many of the behaviours reported in this study align with those documented in the literature (table 4). However, seven strategies in prior reviews were not reported by respondents in this study: drinking sugar-sweetened beverages or consuming foods in place of water; changing agricultural practices; forgoing hygiene; relying on humanitarian assistance; negotiating, stealing or bribing for water; constructing alternative sources or drilling wells and illegally connecting to public water networks (table 4).

**Table 4:** Water insecurity coping strategies reported across HWISE study sites and prior literature reviews

Coping strategies	HWISE study	Venkataramanan *et al* 36	Achore *et al* 34	Majuru *et al* 35
Change food preparation and/or cooking				
Treat water				
Use unsafe water				
Drink sugar-sweetened beverages or consume fruit or other hydrating foods in place of water				
Change agricultural practices				
Forgo hygiene (eg, bathing, hand washing)				
Ask service provider to restore water				
Borrow water				
Borrow money to buy water				
Buy water				
Buy water on credit				
Hire someone to get water				
Work for water or money to buy water				
Collect water from another source				
Rely on humanitarian assistance				
Steal/bribe/negotiate for water				
Change daily routine to acquire water				
Economise water				
Relocate or consider relocating household				
Repair source				
Send children to collect water				
Construct alternative water sources or drill wells				
Create illegal connections to public water networks				
Water harvesting, reliance on rainwater				
Psychosocial coping (suffer, cry, complain, stress, despair)				
Spiritual coping (pray, seek spiritual guidance, get water from places of worship)				
Go without water				
Wait for water to be restored				
Do nothing				

HWISE, Household Water InSecurity Experiences.

#### Water Insecurity Coping Strategies Assessment Toolkit

After consolidating the data set of coping responses and supporting literature, we developed the Water Insecurity Coping Strategies Assessment Toolkit (online supplemental materials 1). This toolkit provides a roster of documented coping strategies with guidance on measuring incidence, prevalence and severity, modelled after the Food Insecurity Coping Strategies Index.^32^

10.1136/bmjgh-2023-013754.supp1 Supplementary data

This toolkit should be paired with an experiential water insecurity scale to assess primary appraisal (ie, perceived harm from water insecurity); for this reason, recall options align with the WISE Scales. The toolkit should also contain a module that assesses secondary appraisal (ie, resources available to buffer against perceived harm from water insecurity).^30^ This would include data on primary and secondary household water sources, time to collect water (roundtrip), household income and water storage capacity.

## Discussion

Using the most comprehensive empirical data set on water insecurity coping strategies collected to date, we sought to characterise coping strategies across a diversity of sites in low-income and middle-income contexts, evaluated associations between reported coping strategies and water insecurity experiences and consolidated coping strategies into a Water Insecurity Coping Strategies Assessment Toolkit.

To the first objective, this study identified 19 distinct coping strategies across 11 sites in 10 low and middle-income countries. Borrowing water was the most common way that households coped with water insecurity. Because this strategy relies on established social networks that can be accessed for resources during times of scarcity,^42^ this suggests that social capital and resource pooling^43^ may be important pathways by which households manage water insecurity. However, when resource borrowers and lenders both experience shock, redistribution is a less effective strategy and can strain socially-reciprocal relationships and hinder long-term resilience.^44^ Future research should map social networks to determine the strengths between water sharing nodes, the threshold of insecurity at which water sharing occurs, expectations of reciprocity and the cumulative impact of water sharing and resource pooling on resilience outcomes.^45 46^

Several psychosocial (eg, anger, fear, suffering) and spiritual (eg, relying on an external spiritual power) behaviours that have been framed elsewhere as consequences of water insecurity were described as responses to having insufficient water or money to buy water, ie coping strategies. Many of these psychosocial responses, specifically suffering, stress and despair, have been well documented in the water literature.^17 47–50^ For example, in Cochabamba, Bolivia, emotional distress (fear, anger, suffering) arose from complex negotiations to obtain water as a product of insufficient water rights.^17^ In *colonias* on the Texas-Mexico border, living with severely constrained water resources resulted in fear and anger.^49^ In a study in Mexico, water insecurity was experienced as ‘suffering’, resulting in a cascade of adverse psychosomatic responses.^50^ In Bangladesh, women reported ‘suffering for water’ due to insufficient access, use and control and ‘suffering from water’ through adverse health outcomes associated with consumption of arsenic-contaminated water.^47^ Conversely, one review indicated that prayers, songs and rituals are portrayed in folklore, ethnography and archaeology as a collective approach to managing distress over low rainfall and drought.^18^ Here, a unique contribution of our inductive approach is highlighting that respondents experiencing water insecurity considered these behaviours to be coping. This illustrates the need for a broader conceptualisation of ‘coping’ that does not necessarily imply success, and the understanding that ‘strategy’ may not imply deliberate agency. It also suggests that further work is needed to understand if strategies are a by-product of chronic resource scarcity or if they serve as a protective mechanism to manage the psychological, social and emotional consequences of water insecurity.

To our second objective, this study provides partial confirmation that water insecurity is associated with particular forms of coping, including borrowing water, using unsafe water and employing psychosocial coping. Because the data are cross-sectional, it is difficult to ascertain the temporality of these relationships, for example, if coping successfully reduces water insecurity. Future studies should seek to evaluate these relationships longitudinally, to understand the characteristics beyond water insecurity experiences that drive these relationships and clarify how coping buffers households against adverse experiences of water insecurity.

To our third objective, we have advanced the measurement of water insecurity coping strategies by outlining guidance on how to systematically assess it. Future work should seek to understand which strategies, if any, afford individuals the potential to meet water needs without endangering livelihoods, social cohesion, well-being and resilience and indicate which strategies pose irreversible harm. Piloting and refining the Water Insecurity Coping Strategies Assessment Toolkit is a first step towards documenting coping and understanding how it contributes to resilience and vulnerability.

### Strengths and limitations

A strength of this study is the wide geographic, economic and ecological scope of the HWISE study data, which is complemented by findings from the literature to maximise diversity of contexts with evidence of coping. Nevertheless, there are a number of cultures and ecologies not represented by our analyses; only one sub-Saharan African site was included, and most sites were urban or peri-urban. Future studies should seek to assess coping in overlooked settings, such as in humanitarian emergencies and in high-income contexts.

A second strength is that this is the first empirical exploration of the relationship between water insecurity and coping. A limitation of this study is that data were cross-sectional, and the open-ended structure of the question did not include a clear recall period nor probing for a more extensive list of all possible coping strategies used. These concerns were acknowledged in a recent paper proposing an update to water insecurity measurement tools.^37^ Here, we address these concerns with a Water Insecurity Coping Strategies Assessment Toolkit (online supplemental materials 1), which we advise using longitudinally in the same context to assess incidence, prevalence, and severity of coping.

Finally, the phrase ‘coping strategy’, although widely used and accepted, may not be thoroughly conceptualised to include individual agency. However, given the noted limitation that data presented here are cross-sectional, we were unable to evaluate adaptation to water insecurity. As our understanding of coping evolves, this nomenclature may be revised. However, it is important to note that coping may not necessarily convey maladaptation.^51^ Studies evaluating coping using the framework we propose should assess resilience and vulnerability to parse these relationships. Future work should also delineate coping from adaptation and management and provide guidance on the appropriate use of these terms for researchers and practitioners.

## Conclusion

Households and individuals rely on a variety of responses to cope with water insecurity; this study captured novel experiences and constructed the first comprehensive toolkit to evaluate coping strategy frequency and severity using the existing literature and empirical findings. This work builds the foundation for future inquiry on water insecurity coping. The proposed Water Insecurity Coping Strategies Assessment Toolkit should be used across a variety of settings to assess its suitability for measuring coping strategies across a wide range of geographic, ecological and cultural contexts.

## Data Availability

Data are available upon reasonable request. Data are available upon request from the HWISE Research Coordination Network.
