# Supplementary material for: Toward a more systematic understanding of water insecurity coping strategies: insights from 11 global sites
Source: BMJ Glob Health. 2024 May 31;9(5):e013754. doi: 10.1136/bmjgh-2023-013754 (PMC11149151; doi:10.1136/bmjgh-2023-013754)
Supplement: Supplementary data [file bmjgh-2023-013754supp001.pdf]

SUPPLEMENTAL MATERIAL 1

WATER INSECURITY COPING STRATEGIES ASSESSMENT TOOLKIT

STEP ONE: The first step is to ascertain coping strategies used in specific contexts, and the frequency by which they occur. Focus group discussions (FGDs) should be held to collect this information.(1) Three to six FGDs(2) with 10-12 interviewees should capture 90% of themes.(3) FGDs should be oversampled for women in areas where they are disproportionately responsible for intrahousehold water management,(4) paralleling methodology outlined by Maxwell et al., 2008.(5)

During FGDs, coping strategies should be free-listed in response to the question “what do you do when you do not have enough water or money to buy water?” using the Water Insecurity Coping Strategies Assessment Focus Group Discussion Guide to facilitate. It should be clearly communicated that the strategies should only be those used in response to water insecurity or water-related shocks (e.g., flooding, drought) and not as part of routine household water management. All strategies mentioned during free-listing should be followed up with the question, “have you used this strategy in the past four weeks?” Enumerators should denote all of these strategies. Following free-listing, enumerators should ask “In the last four weeks, have you ever [coping strategies listed in the roster] when you did not have enough water or money to buy water?” Only coping strategies listed in the roster that have not been mentioned (c.f., Water Insecurity Coping Strategies Assessment Focus Group Discussion Guide) should be queried on. Enumerators should record the number of respondents who said ‘yes’ to the question.

STEP TWO: After noting all of the coping strategies that have been used in the past four weeks, respondents should be asked to rank items in terms of perceived frequency, coming to consensus on the relative frequency that each strategy is used in the context.

STEP THREE: Once all of the coping strategies have been listed, respondents should rank items in terms of their relative severity (i.e., how severe is the water insecurity situation to necessitate use of the strategy) from most to least severe.(5) Severity may be perceived differently based on the context and respondents. Enumerators should start by asking FGD respondents how they conceptualize severity. After the items have been ranked, respondents should group responses into severity categories; not severe (scored 1), moderately severe (scored 2), severe (scored 3), and extremely severe (scored 4).(5)

STEP FOUR: The resultant shortlist of the 10-15 most frequently reported coping strategies should be selected and written down in the Water Insecurity Coping Strategies Assessment Pilot Survey Tool. This tool should be piloted broadly among a randomly selected sample in the context where FGDs were conducted to assess the prevalence, frequency, and severity of coping strategies. Severity should be pre-populated according to responses reported in FGDs (e.g., strategies identified as ‘extremely severe’ should be scored as 4 in the pilot survey tool). Frequency should be assessed using the question “If you responded yes, how often in the last four weeks have you [coping strategy]?” Response options mirror the WISE Scales; never (0 times, scored 0), rarely (1-2 times, scored 1), sometimes (3-10 times, scored 2), often (11-20 times, scored 3), and always (more than 20 times, scored 4).(6) Frequency should be multiplied by severity response to create a weighted score.(5)

This toolkit should be paired with an experiential water insecurity scale to assess primary appraisal (i.e., perceived harm from water insecurity).(7) For this reason, recall options align with the WISE Scales. The toolkit should also contain a module that assesses secondary appraisal (i.e., resources available to buffer against perceived harm from water insecurity).(7) This would include data on primary and secondary household water sources, time to collect water (roundtrip), household income, and water storage capacity.

Water Insecurity Coping Strategies Assessment Focus Group Discussion Guide

**ROSTER OF COPING STRATEGIES**	**In the last four weeks, have you ever...when you did not have enough water or money to buy water?**<br><br>**Record yes/no and number of people who used this strategy**		**Please rank all selected strategies from most to least frequent**	**Please rank all selected strategies from most to least severe**	**Severity group**<br><br>1...Not Severe<br>2...Moderately Severe<br>3...Severe<br>4...Very severe
	**Yes/No**	**Number of responses**			
Skipped cooking or prepared foods that use less water?					
Skipped washing your dishes or used recycled water to wash dishes?					
Treated your water (chemicals, boiling, filtering)?					
Drank or cooked with water that you thought was unsafe or might make you sick?					
Drank sugar-sweetened beverages (juice, soda, others) in place of water?					
Consumed fruit or other hydrating foods in place of water?					
Changed agricultural practices (water crops or animals less, change timing of planting, sell animals or harvest sooner, use dirty or recycled water for plants or animals)?					
Gone without washing your hands, body, or skipped other hygiene needs?					
Used dirty or recycled water to wash hands, body, or for other hygiene activities?					
Skipped washing your clothing or used recycled water to wash clothing?					

Used less water or worse quality water for prayer/religious purposes?					
Asked a service provider to restore water?					
Borrowed water from another person?					
Borrowed money from another person to buy water?					
Purchased water from a vendor?					
Purchased water using credit?					
Asked someone to get water for you (paid)?					
Asked someone to get water for you (unpaid)?					
Worked for water or money to buy water?					
Collected water from a source that isn't the one you typically use?					
Relied on humanitarian assistance (i.e., from WFP or an NGO, or others) for water?					
Stolen, negotiated, or bribed someone to get water?					
Traded sex or sexual favors for water or to skip the water queue?					
Changed your daily routine to get water (e.g., woke up earlier than planned, collected water during the night or in the dark)?					
Stored or used stored water?					
Considered relocating your household because of water?					
Relocated your household because of water?					
Repaired your water source?					

Sent children from the household to collect water (e.g., from neighbors, from school, from family, friends, or other sources)?					
Prioritized water needs (for drinking, bathing, etc.) for some household members over others?					
Sent some family members to live in other households, placed children for adoption or foster, or abandoned household members for water?					
Constructed alternative water sources (drilled a well, built a pond)?					
Connected illegally to piped water networks?					
Gathered rainwater?					
Cried, suffered, complained, felt stress or despair because of water?					
Prayed for water, sought spiritual guidance, or gotten water from a place of worship (church, mosque)?					
Gone without water?					
Waited for water to be restored?					
Done nothing about your water situation?					
**Additional or context-specific strategies (please specify)**					

Water Insecurity Coping Strategies Assessment Pilot Survey Tool

**Coping Strategies (CS) Shortlist**	**In the last four weeks, have you ever...when you did not have enough water or money to buy water how often have you...?**<br><br>0...Never (0 times)<br>1...Rarely (1-2 times)<br>2...Sometimes (3-10 times)<br>3...Often (11-20 times)<br>4...Always (more than 20 times)	**Severity group**<br><br>1...Not Severe<br>2...Moderately Severe<br>3...Severe<br>4...Very severe	**Weighted score (frequency x severity)**
CS 1:			
CS 2:			
CS 3:			
CS 4:			
CS 5:			
CS 6:			
CS 7:			
CS 8:			
CS 9:			
CS 10:			
CS 11:			
CS 12:			
CS 13:			
CS 14:			
CS 15:			
**TOTAL**			

1. Maxwell D, Watkins B, Wheeler R, Collins G. The Coping Strategies Index: A tool for rapidly measuring food securty and the impact of food aid programmes in emergencies. In Tivoli; 2003. Available from: http://www.fao.org/3/a-ae513e.pdf

2. Guest G, Namey E, McKenna K. How Many Focus Groups Are Enough? Building an Evidence Base for Nonprobability Sample Sizes. Field Methods. 2017 Feb;29(1):3–22.

3. Breen RL. A Practical Guide to Focus-Group Research. Journal of Geography in Higher Education. 2006 Nov;30(3):463–75.

4. Ray I. Women, Water, and Development. Annual Review of Environment and Resources. 2007 Nov 1;32(1):421–49.

5. Maxwell D, Caldwell R. The Coping Strategies Index: A tool for rapid measurement of household food security and the impact of food aid programs in humanitarian emergencies. [Internet]. Cooperative for Assistance and Relief Everywhere, Inc. (CARE).; 2008 p. 52. Available from: https://www.fsnnetwork.org/sites/default/files/coping_strategies_tool.pdf

6. Young SL, Collins SM, Boateng GO, Neilands TB, Jamaluddine Z, Miller JD, et al. Development and validation protocol for an instrument to measure household water insecurity across cultures and ecologies: the Household Water InSecurity Experiences (HWISE) Scale. BMJ Open. 2019 Jan;9(1):e023558.

7. Lazarus RS, Folkman S. Stress, appraisal, and coping. 10. print., [Nachdr.]. New York, NY: Springer; 2006. 445 p.
